# Study on Nanoporous Graphene-Based Hybrid Architecture for Surface Bonding

**DOI:** 10.3390/nano12142483

**Published:** 2022-07-20

**Authors:** Xiaohui Song, Mingxiang Chen, Jingshuang Zhang, Rui Zhang, Wei Zhang

**Affiliations:** 1 Institute of Applied Physics, Henan Academy of Science, Zhengzhou 450008, China; 13513808093@163.com; 2 School of Mechanical Science and Engineering, Huazhong University of Science and Technology, Wuhan 430074, China; chimish@163.com; 3 School of Mechanical Engineering, Zhengzhou University, Zhengzhou 450001, China; zhangjingshuang@hniot.com (J.Z.); lyzr@zzu.edu.cn (R.Z.)

**Keywords:** surface bonding, nanoporous graphene

## Abstract

Graphene-copper nanolayered composites have received research interest as promising packaging materials in developing next-generation electronic and optoelectronic devices. The weak van der Waal (vdW) contact between graphene and metal matrix significantly reduces the mechanical performance of such composites. The current study describes a new Cu-nanoporous graphene-Cu based bonding method with a low bonding temperature and good dependability. The deposition of copper atoms onto nanoporous graphene can help to generate nanoislands on the graphene surface, facilitating atomic diffusion bonding to bulk copper bonding surfaces at low temperatures, according to our extensive molecular dynamics (MD) simulations on the bonding process and pull-out verification using the canonical ensemble (NVT). Furthermore, the interfacial mechanical characteristics of graphene/Cu nanocomposites can be greatly improved by the resistance of nanostructure in nanoporous graphene. These findings are useful in designing advanced metallic surface bonding processes and graphene-based composites with tenable performance.

## 1. Introduction

Surface bonding is an essential step in three-dimensional electronic packaging, as it provides mechanical support, heat transfer, and electrical integration [1]. A low-temperature bonding technology and reliable connection interface greatly influence electronic systems’ performance and service life, especially when packaging density dramatically increases with device scaling [2,3]. Traditional surface bonding techniques in electronic assembly rely on high-temperature processes such as reflow soldering [4,5] and thermo-compression bonding [6], which can lead to undesirable thermal damage, toxic solder materials pollution, and a thermal mismatch at the bonding interface. Recently various nanometal materials such as metal nanowires, nanoparticles, and nanocones-based surface bonding are being studied to lower the bonding temperature and pressure [7,8,9,10]. However, these bonding technologies introducing low deformation resistance joints have been entangled in thermo-mechanical stresses and aging degradation issues, limiting their reliability. Therefore, it is necessary to develop high stretch and shear deformation resistance of interconnection materials architecture and a low-temperature bonding process for a complex interface of three-dimensional packaging structures.

Since its isolation as a two-dimensional (2D) system, graphene-copper nanolayered composites have been widely acclaimed and proved to be the effective alternatives to pure metal materials owing to their outstanding mechanical and heat transfer properties [11,12,13,14,15], showing great promise for next-generation electronic and optoelectronic device packaging. However, the reinforcing effect of graphene is limited by vdW non-bonded interfacial interaction between graphene and Cu, resulting in a low load transfer rate from Cu matrix to graphene during mechanical deformation [16]. To pave the way toward practical applications, a variety of approaches have been developed, such as chemical functionalization and metal coating [17,18]. The controllable and precise solution that uses graphene-metal composites as bonding layers remains a major challenge, despite considerable progress.

Here, we report a simple new Cu-nanoporous graphene-Cu based bonding technology that is of low bonding temperature and high reliability. We transfer nanoporous graphene to the copper surface and form copper nanoparticles on nanopores by depositing copper atoms before thermocompression bonding. Since the copper nanoparticles deposited in the nanopores form a metal bond connection with the copper bonding surface, they effectively enhance the interfacial connection and, consequently, lead to higher interfacial shear strength between graphene and Cu surface. At the same time, the metal nanoparticles contribute to solid-state diffusion and intermixing of surface atoms between the interfaces at low temperatures. The present study aims to quantitatively investigate the above-mentioned process. Comprehensive molecular dynamic simulations are carried out, and the results reveal that the copper-nanoporous graphene-copper sandwich structure can be bonded at low temperature by the thermocompression method, and the interfacial shear strength of the interface can be significantly increased by nanoporous-nanometal particles hybrid structure.

## 2. Computational Methods

**Figure 1:**
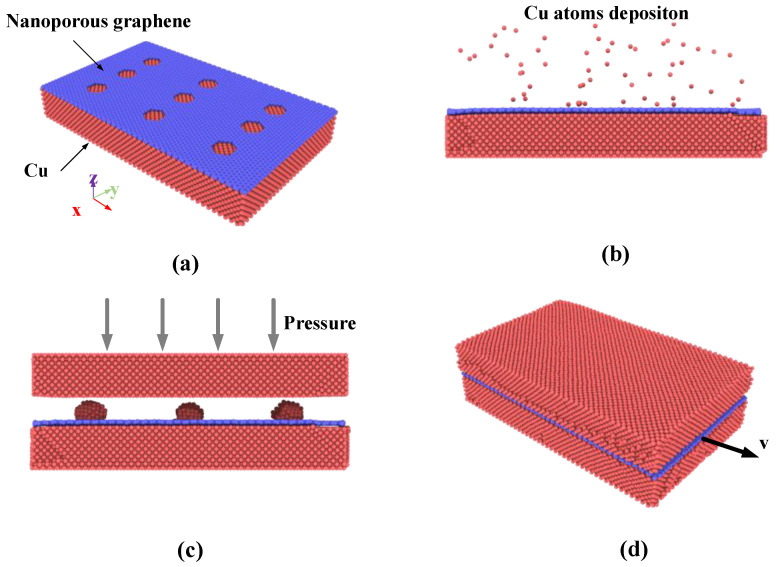
Atomic configurations. (**a**) Cu-nanoporous graphene composite model; (**b**) Cu atoms deposition onto the nanoporous graphene surface; (**c**) Cu-nanoporous graphene-Cu thermocompression bonding; (**d**) Pull-out simulation model.

For molecular dynamics simulation of Cu-nanoporous graphene-Cu bonding process and the reinforcing effect evaluation, the setup of the simulations performed in the present work is shown in Figure 1. Initially, the composite model is constructed by covering single-layer graphene with nine nanopores onto the single crystal copper surface that contains 34,924 atoms with the size of 161.78 Å × 99.65 Å× 23.35 Å, as shown in Figure 1a. The boundary condition for the simulation box is periodic in the *x* and *y* directions while the nonperiodic boundary condition is applied along the *z*-direction. The equilibrium structures are achieved using the canonical ensemble (NVT) [19] of the constant volume and temperature for 20 ps with a time step of 0.5 fs.

Next, the bottommost two layers are fixed. The other atoms above the fixed layers are defined as temperature control layers that control the system temperature using the canonical ensemble (NVT), and the time step is set to 0.5 fs. In the condition of copper sputtering deposition simulation, the deposition rate of incident Cu atoms is 0.25 atoms/ps. The coordinates of incident Cu atoms were distributed randomly within the defined insertion volume, which is 50 nm above the graphene surface, as shown in Figure 1b. In each simulation, after all Cu atoms are released, a relaxation process of 2500 ps is conducted to enable the deposited atoms to reach full thermal equilibrium.

Then the bonding model is constructed by introducing a single crystal copper cell of the same size as the previous composite model as shown in Figure 1c. The distance between the two surfaces is set to nm. The uppermost two atomic layers are set as a fixed layer to produce the pressure of the system, and five atomic layers connected to the fixed layers are set up as temperature-controlled layers using the canonical ensemble (NVT). The other free copper atoms and nanoporous graphene at the bonding interface use the micro-canonical ensemble (NVE) of the constant volume and energy with a time step of 0.5 fs. During the first stage of simulation, no pressure is loaded on the fixed layer in 2 ns to reduce internal stress and make the model be in a steady state at a certain temperature. The pressure is loaded on the upper fix layer for 4 ns. At last, the pressure is unloaded and the model holds for 5 ns.

Finally, the pull-out simulation is used to investigate the interfacial shear strength of Cu nanocomposite. During the pull-out simulations, the upmost two layers and the bottommost two layers of Cu atoms are fully fixed. The pull-out simulations using the canonical ensemble (NVT) are performed by applying a constant velocity to the graphene along the *x*-direction until it is completely pulled out from the bonding interface as shown in Figure 1d. To ensure that the model is fully relaxed in each step and eliminate the effect of velocity on the pull-out force, a relatively low velocity of 0.8 × 10^−5^ Å/fs is adopted to 245 atoms treated as a rigid body on the rightmost side of the graphene. A temperature of 300 K is employed for all simulations, and a time step of 0.5 fs is adopted throughout the whole simulation process. The boundary condition for the simulation box is periodic in the *y* directions while the nonperiodic boundary condition is applied along the *x* and *z* directions.

The embedded atomic method (EAM) [20] models successfully used in modeling various bulk metals are used to describe the Cu-Cu interactions. For the vdW interactions between the graphene surface and Cu atoms, the Lennard–Jones (LJ) pair potential involving nonbonded long-range interactions is used with parameters σ = 3.0825 Å, ε = 0.02578 eV [21], and a cutoff radius = 4 σ. Our simulations are based on the massively parallel LAMMPS code [22]. The visualization is based on OVITO [23].

## 3. Results and Discussion

**Figure 2:**
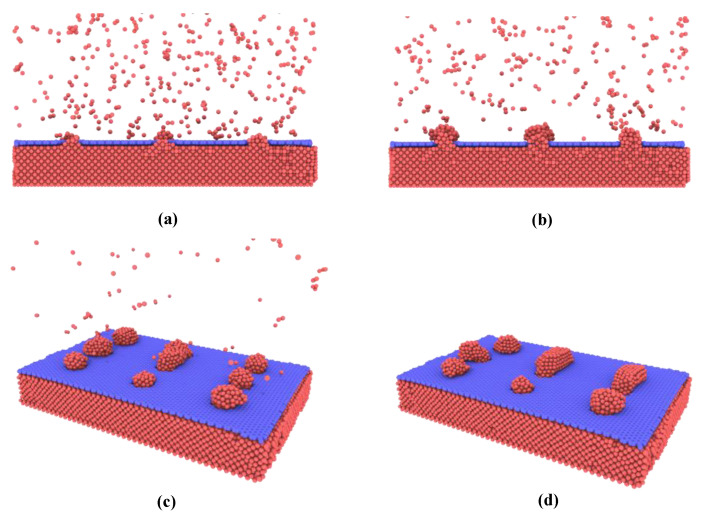
Atomic configurations of Cu atoms deposition onto the graphene at various stages. (**a**) Filling of nanopores at the initial stage; (**b**) Insular growth to nanoislands; (**c**) Further growing of nanoislands; (**d**) Joining with neighboring nanoislands.

First, the Cu atoms deposition onto the nanoporous graphene surface is investigated. As shown in Figure 2a, in the initial state, the Cu atoms prefer to deposit in the nanoporous graphene and form metallic connections with the attached copper surface until the porous are fully filled. The reason for this is that Cu-Cu interactions are stronger than Cu-graphene. More copper atoms are then deposited onto the copper structure in the nanopores and gradually grow to form nanoislands. The growth pattern observed in this simulation approximates the insular growth shown in Figure 2b. When the nanoislands reach a certain size, they join with neighboring nanoislands to form larger ones as shown in Figure 2c,d.

**Figure 3:**
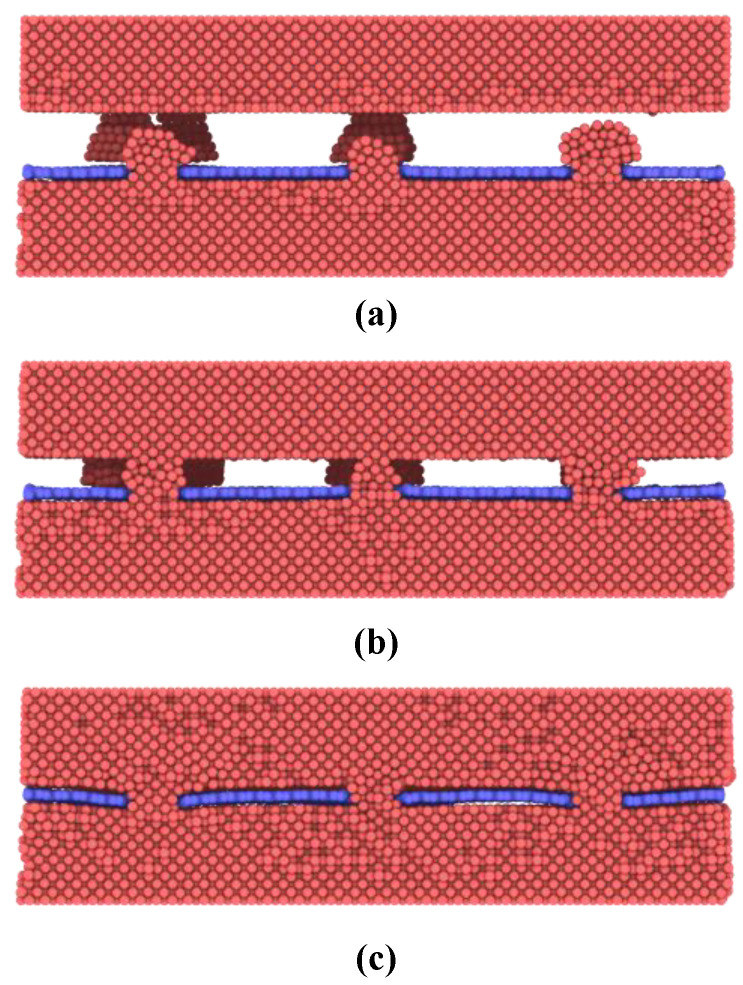
Cross-section configurations of Cu-nanoporous graphene-Cu during the bonding process at various stages. (**a**) Contacting of bonding surface; (**b**) Compression deformation of the nanoislands; (**c**) Final bonding structure.

Then thermocompression bonding simulations are performed to exploit the bonding mechanisms of nanoporous graphene-based hybrid architecture. A constant temperature of 300 K and pressure of 0.5 MPa are applied to the model. In the initial state, two contact interfaces form between nanoislands and the bulk copper surface as shown in Figure 3a. Compression deformation of the nanoislands increases until the bulk copper surface and the graphene are joined together as shown in Figure 3b,c. In this process, the nanoislands are gradually dispersed on the graphene surface and fill the interface while atomic diffusion occurs with the bulk copper surface. During this state, nanoislands undergo a typical process from elastic deformation to plastic deformation, and the crystal structure of nanoislands has been mostly destroyed. As a result, more and more active copper atoms on the nanoislands surface make contact with the bonding surface to form metallic bonding connections with time increasing.

**Figure 4:**
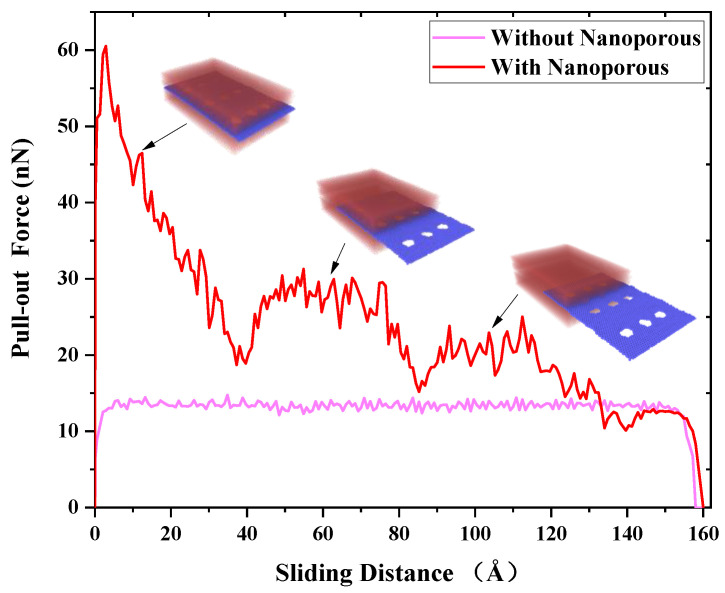
The pull-out force changes as a function of the sliding distance for the graphene/Cu composite with and without nanoporous.

Pull-out simulations of the nanoporous graphene from the metal matrix are used to investigate the reinforcing mechanisms of Cu-nanoporous graphene-Cu nanocomposites. Along the *x*-axis, a constant velocity is delivered to the graphene. Figure 4 shows the pull-out force for graphene/Cu composites with and without nanoporous surfaces in terms of sliding distance. The atomic configurations during the nanoporous pull-out process are depicted in the insets of Figure 4. The pull-out force without nanoporous grows fast until the sliding distance reaches roughly 8 Å as illustrated in Figure 4. After this, it swings at around 13 nN for the next 140 Å. The pull-out force then steadily declines until graphene is totally pulled out in about the last 10 Å of sliding distance. In this condition, the van der Waals interaction between the graphene surface and the copper atoms at the interface dominates the pull-out force. The pull-out force with nanoporous, on the other hand, rises quickly to about 60 nN at the start of the pull-out process, nearly six times higher than without nanoporous. Copper atoms in nanopores and their van der Waals contact with the graphene surface are credited with resisting the pull-out motion of nanoporous graphene. In particular, the graphene pull-out process requires breaking through the resistance of the nanostructures in the pores, which greatly increases the pull-out force. Therefore, much larger pull-out force is generated in graphene. As a result, a larger pull-out force is needed for the nanoporous graphene, giving rise to better interfacial properties than that of pure graphene.

**Figure 5:**
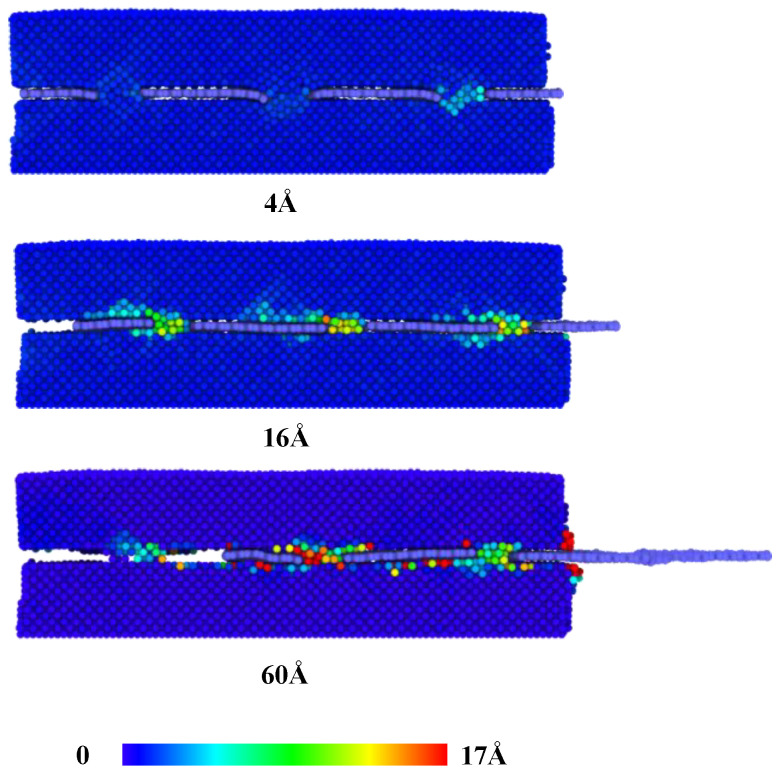
The displacement of copper atoms at the interface of graphene/Cu nanocomposites with various sliding distances.

When the sliding distance is larger than 4 Å, the force decreases to about 18 nN when the first column of nanoporous is fully pulled out. Figure 5 presents the displacement of copper atoms at the interface of graphene/Cu nanocomposites for this process. As the sliding distance increases, the atoms in the nanoporous are largely deformed until they move along with the graphene. The fluctuation of the pull-out force corresponds to the deformation-movement process of copper structures in nanoporous. Consequently, van der Waals interactions play an increasingly important role, and the copper atoms in nanopores are becoming less resistant to graphene. Next, as the sliding distance increases, there are two processes of increasing and decreasing pull-out force until it becomes 0. It is noted that these two processes occur after the first and second columns of nanoporous are pulled out, respectively, as shown in Figure 3. Since the graphene is regarded as a flexible body during the pull-out process, during the constant velocity pull-out process, the outer porous are subjected to more deformation, which makes the unpulled part move relatively slowly, so the atoms in the nanoporous move more slowly, and the deformation effect of the structure makes it have more resistance. Therefore, the pull-out force appears to increase.

**Figure 6:**
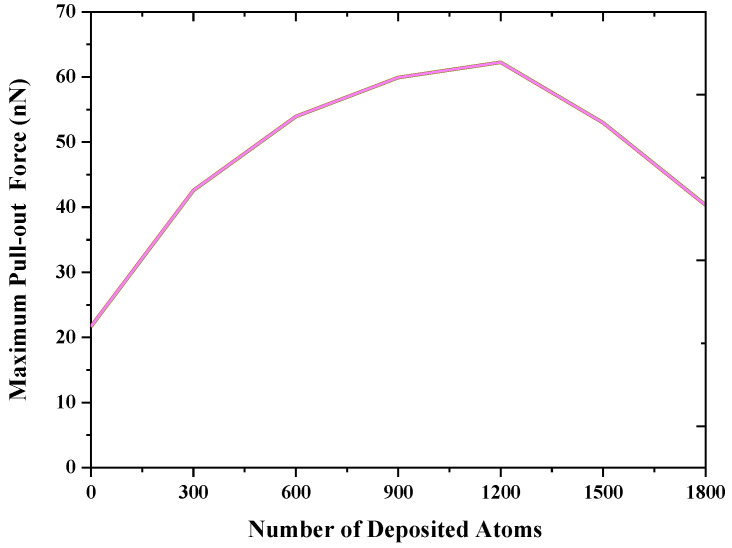
The maximum pull-out force changes as a function of the number of deposited copper atoms for the graphene/Cu composite.

As mentioned above, the deformation resistance of the nanostructures obtained by depositing copper atoms in nanoporous is the key to enhancing the interfacial mechanical properties of composites. For this purpose, the effect of the number of deposited copper atoms on the pull-out force is investigated in the present work. Figure 6 shows the maximum pull-out force change as a function of the number of deposited copper atoms. A larger maximum pull-out force can be observed as-deposited atoms onto the graphene increase. The reason for this is that more deposited atoms help fill the nanopores and form nanoislands to form diffusion bonds with the bulk copper bonding surface. The nanostructures in nanoporous can increase the deformation resistance effect, consequently leading to improved interfacial mechanical properties. However, when the deposited atoms exceed 1200, the maximum pull-out force decreases instead, mainly because the oversized nanoisland structure is not sufficiently deformed in the bonding, which makes the bonding surface not fully in contact with the graphene surface and reduces the van der Waals force interaction between copper and graphene surface.

**Figure 7:**
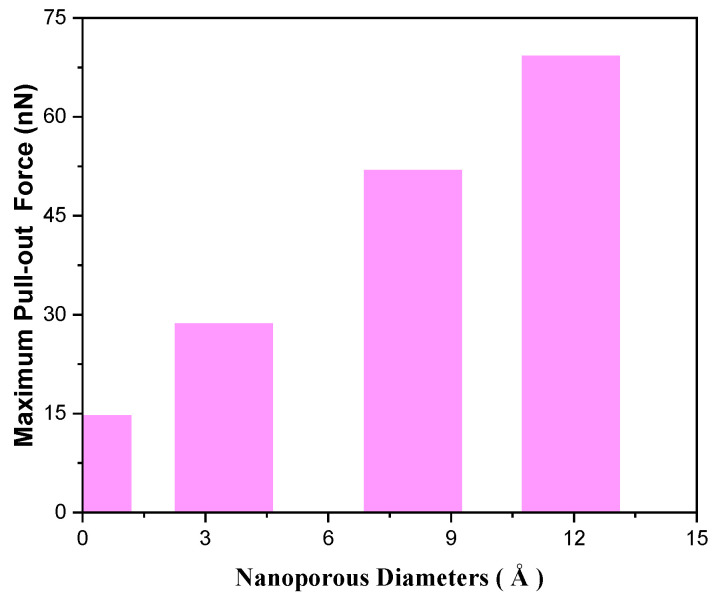
The maximum pull-out force changes vs. nanoporous diameters.

The influence of nanoporous diameters on the reinforcing effect of graphene/Cu nanocomposites is also further studied. We have considered three different nanoporous diameters: small (3.45 Å), medium (8.07 Å), and large (11.93 Å). The number of deposited atoms is 500. As shown in Figure 7, the larger the nanoporous, the greater the maximum pull-out force. The reason is that larger nanoporous helps to form bigger and stronger nanostructures in the nanoporous, which are not deformable and require greater pull-out forces to destroy the nanostructures. However, larger holes make the effective area of graphene reduced, which may affect the ability of graphene to perform thermal and electrical transport functions.

## 4. Conclusions

In this study, we report a novel Cu-nanoporous graphene-Cu based bonding technology that is of low bonding temperature and high reliability. The process of depositing copper atoms and thermocompression bonding has been numerically investigated by employing MD simulations. Numerical results show that deposition of copper atoms onto nanoporous graphene can help to generate nanoislands on the graphene surface, facilitating atomic diffusion bonding to bulk copper bonding surfaces at low temperatures. Moreover, the resistance of nanostructure in the nanoporous can dramatically improve the interfacial mechanical properties of graphene/Cu nanocomposites. It is worth mentioning that an increase in the number of deposited atoms enhances the maximum pull-out force, but too many atoms deposited will reduce the interfacial strength. While larger nanoporous helps to form bigger and stronger nanostructures in the nanoporous, which are not deformable and require greater pull-out forces to destroy the nanostructures. Decidedly, the research findings of this study provide a feasible and facile route for low-temperature metal surface bonding with a high-performance metal nanocomposites interface layer reinforced by nanoporous graphene.

## Data Availability

The raw/processed data required to reproduce these findings cannot be shared at this time as the data also forms part of an ongoing study.

## References

[1] Lancaster A., Keswani M. (2018). Integrated circuit packaging review with an emphasis on 3D packaging. Integration.

[2] Han H., Lee C., Kim Y., Lee J., Kim R., Kim J., Yoo B. (2021). Cu to Cu direct bonding at low temperature with high density defect in electrodeposited Cu. Appl. Surf. Sci..

[3] Panigrahy A.K., Chen K.-N. (2018). Low Temperature Cu–Cu Bonding Technology in Three-Dimensional Integration: An Extensive Review. J. Electron. Packag..

[4] Wang J., Wang Q., Wu Z., Tan L., Cai J., Wang D. (2017). Plasma combined self-assembled monolayer pretreatment on electroplated-Cu surface for low temperature Cu–Sn bonding in 3D integration. Appl. Surf. Sci..

[5] Liang L., Zhang J., Xu Y., Zhang Y., Wang W., Yang J. (2018). The effect of pressure and orientation on Cu-Cu3Sn interface reliability under isothermal ageing and monotonic traction via molecular dynamics investigation. Mater. Des..

[6] Shie K.-C., Gusak A.M., Tu K.N., Chen C. (2021). A kinetic model of copper-to-copper direct bonding under thermal compression. J. Mater. Res. Technol..

[7] Alian A.R., Ju Y., Meguid S.A. (2019). Comprehensive atomistic modeling of copper nanowires-based surface connectors. Mater. Des..

[8] Song X., Wu S., Zhang R. (2021). Computational Study on Surface Bonding Based on Nanocone Arrays. Nanomaterials.

[9] Mou Y., Cheng H., Peng Y., Chen M. (2018). Fabrication of reliable Cu-Cu joints by low temperature bonding isopropanol stabilized Cu nanoparticles in air. Mater. Lett..

[10] Zhang S., Xu X., Lin T., He P. (2019). Recent advances in nano-materials for packaging of electronic devices. J. Mater. Sci. Mater. Electron..

[11] Kim Y., Lee J., Yeom M.S., Shin J.W., Kim H., Cui Y., Kysar J.W., Hone J., Jung Y., Jeon S. (2013). Strengthening effect of single-atomic-layer graphene in metal–graphene nanolayered composites. Nat. Commun..

[12] An Z., Li J., Kikuchi A., Wang Z., Jiang Y., Ono T. (2019). Mechanically strengthened graphene-Cu composite with reduced thermal expansion towards interconnect applications. Microsyst. Nanoeng..

[13] Peng W., Sun K. (2020). Effects of Cu/graphene interface on the mechanical properties of multilayer Cu/graphene composites. Mech. Mater..

[14] Wang H., Leong W.S., Hu F., Ju L., Su C., Guo Y., Li J., Li M., Hu A., Kong J. (2018). Low-Temperature Copper Bonding Strategy with Graphene Interlayer. ACS Nano.

[15] Kakanakova-Georgieva A., Gueorguiev G., Sangiovanni D.G., Suwannaharn N., Ivanov I.G., Cora I., Pécz B., Nicotra G., Giannazzo F. (2020). Nanoscale phenomena ruling deposition and intercalation of AlN at the graphene/SiC interface. Nanoscale.

[16] Hidalgo-Manrique P., Lei X., Xu R., Zhou M., Kinloch I.A., Young R.J. (2019). Copper/graphene composites: A review. J. Mater. Sci..

[17] Montazeri A., Panahi B. (2018). MD-based estimates of enhanced load transfer in graphene/metal nanocomposites through Ni coating. Appl. Surf. Sci..

[18] Zhao S., Zhang Y., Yang J., Kitipornchai S. (2021). Significantly improved interfacial shear strength in graphene/copper nanocomposite via wrinkles and functionalization: A molecular dynamics study. Carbon N. Y..

[19] Hickman J., Mishin Y. (2016). Temperature fluctuations in canonical systems: Insights from molecular dynamics simulations. Phys. Rev. B.

[20] Hao H., Lau D. (2017). Atomistic modeling of metallic thin films by modified embedded atom method. Appl. Surf. Sci..

[21] Long X.J., Li B., Wang L., Huang J., Zhu J., Luo S. (2016). Shock response of Cu/graphene nanolayered composites. Carbon N. Y..

[22] Plimpton S. (1995). Fast Parallel Algorithms for Short-Range Molecular Dynamics. J. Comput. Phys..

[23] Stukowski A. (2009). Visualization and analysis of atomistic simulation data with OVITO–the Open Visualization Tool. Model. Simul. Mater. Sci. Eng..

